# Sarafu Community Inclusion Currency 2020–2021

**DOI:** 10.1038/s41597-022-01539-4

**Published:** 2022-07-20

**Authors:** Carolina E. S. Mattsson, Teodoro Criscione, William O. Ruddick

**Affiliations:** 1 Leiden Institute of Advanced Computer Science, Leiden University, Leiden, Netherlands; 2 Department of Network and Data Science, Central European University, Vienna, Austria; 3 Freiburg Institute for Basic Income Studies, University of Freiburg, Freiburg, Germany; 4 Grassroots Economics Foundation, Kilifi, Kenya

**Keywords:** Economics, Developing world, Scientific data

## Abstract

We describe a dataset of account information and detailed transaction records for a digital complementary currency in Kenya. This “Sarafu system” initially encompassed several local, physical community currencies, which began transitioning to a feature-phone mobile interface in 2017. One unit of “Sarafu” is roughly equivalent in value to a Kenyan shilling. The published data includes anonymized account information for around 55,000 users and records of all Sarafu transactions conducted from January 25, 2020 to June 15, 2021. Transactions totaling around 300 million Sarafu capture various economic and financial activities such as purchases, transfers, and participation in savings and lending groups. So-called “chamas” are key to the operation of the Sarafu system and many such groups are labeled in the data. Describing this data contributes to research on the operation of community currencies, monetary systems, and economic networks in marginalized, food insecure areas. The observation period includes the first year of the COVID-19 pandemic and several documented pilot projects and interventions.

## Abstract (summary)

**Table:** 

Measurement(s)	Payment
Technology Type(s)	Monitoring
Sample Characteristic - Organism	Homo sapiens
Sample Characteristic - Environment	Complementary currency system
Sample Characteristic - Location	Kenya

## Background & Summary

Complementary currencies are mediums of exchange that circulate in parallel to national currencies based on collective agreements among users, such as cryptocurrencies, community currencies, and time banks^1–4^. Community currency systems generally serve a specific local area or region, often with positive social and economic impacts^3,5–10^. Grassroots Economics (GE) is a non-profit foundation based in Kenya specializing in local, bottom-up, economic development projects. Since 2015, GE has been supporting several Kenyan communities in designing their own local currency systems. Today, these operate via a feature-phone mobile interface as so-called Community Inclusion Currencies (CICs) managed using custom-built open source software (see Code Availability).

The “Sarafu system” is our collective term for the currencies managed by GE, from the Kiswahili word for “currency”. By the end of 2019, the system included 12 different community currencies: 8 in urban or periurban areas and 4 in rural areas, spanning a large geographic area from Mombasa to Nairobi^10–12^. On January 25, 2020, these precursor currencies were folded into a single CIC called simply “Sarafu”. The Sarafu CIC 2020–2021 data is comprised of digital records collected in the routine administration of Sarafu, from January 25, 2020 to June 15, 2021^13^. It was published by GE as a part of its broader mission, made available via the UK Data Service ReShare repository with suitable limitations on access and reuse.

Here, we describe the Sarafu CIC 2020–2021 data. The Data Records section details the “transaction dataset” of accounting records and the “user dataset” of anonymized information about account holders. The Methods section explains how these records came to be, including the different account types, transaction types, and currency management activities. Over the observation period, the number of registered accounts grew from 8,354 to around 55,000. Transactions capturing purchases, transfers, and participation in local savings and investment groups total around 300 million Sarafu or $2.8 million US Dollars.

We also provide necessary context for researchers. For example, informal financial institutions used to pool, lend, or invest the savings of its members^14,15^ are widespread in Kenya^16^ and feature prominently in the Sarafu system^11,17^. Field staff at GE verify the activities of savings and investment groups—“chama” is the local term, Kiswahili for “group”. Moreover, GE concentrates its efforts in marginalized, food insecure areas. Partner organizations have included the International Federation of Red Cross and Red Crescent Societies, the Danish, the Kenyan, and the Norwegian Red Cross, the Norwegian Government, the DOEN Foundation, the United Nations International Children’s Emergency Fund (UNICEF), the World Food Programme (WFP), and the Deutsche Gesellschaft für Internationale Zusammenarbeit (GIZ). Recognized chamas received various forms of donations over the observation period, which includes the first year of the COVID-19 pandemic. Contextual details are provided in Usage Notes, documenting several development, humanitarian, and experimental interventions.

This dataset will contribute to applied research on community currencies, humanitarian aid, and economic development. Ussher *et al*.^18^ discuss practical challenges facing modern models of humanitarian and development aid; they analyze a version of the dataset described here to study the potential of CICs to improve cash transfer programs and develop local markets. Community currencies have a long history of meeting liquidity needs in local economies, and have been implemented all over the world^19–23^. However, transaction data is only rarely available for research on community currencies and their effects^1,3,24–26^. That the observation period extends well into the COVID-19 pandemic is especially relevant, as community currencies tend to have higher utility during economic and/or financial crises^1,3,4,19,25,27^. More broadly, this dataset serves as a detailed account of an innovative digital currency system of relevance to research topics like digital and financial inclusion^28–32^ or informal credit and insurance arrangements^15,26,33,34^.

This dataset will also contribute to basic research on monetary systems, networked walk processes, and on the formation, structure, and dynamics of social, economic, and financial networks. Empirical research on money and payments are typically studied via surveys or measures constructed from macroeconomic aggregates^35–38^. Datasets such as the one described in this paper allow for empirical observation at higher granularity^1,39–42^ with the potential to inform modelling of currencies and payment systems^43–47^. Financial transactions are also an example of real-world walk processes on networks; this dataset will support the development of novel techniques for temporal network analysis^32,48^. It will be of particular interest for studying economic networks, where transaction data could be used to explore the relation between network topology, trading dynamics, and allocation of resources^34,49–54^.

## Methods

Digital records of transactions and information on account holders were collected in the routine administration of the Sarafu system. In this section, we describe the payment platform and the record-keeping systems active during the observation period. Information about account holders was extracted from the administrative database on June 15, 2021, as were all Sarafu transactions conducted between January 25, 2020 and June 15, 2021. Grassroots Economics (GE) anonymized the dataset and has made it available with suitable limitations on access and reuse via the UK Data Service ReShare repository^13^.

### Payment platform

Users interact with the Sarafu community currency over a USSD (Unstructured Supplementary Service Data) interface, secured behind an authentication code. The core functions of the interface are checking the account balance, making Sarafu transactions to other accounts, and updating account information. These are implemented via feature-code menus accessible from most mobile phones over regular cellular service, that is, USSD does not require an internet connection. For details about the USSD interface, we refer to the documentation folder accompanying the dataset^13^ and Fig. 2 of Ussher *et al*. (2021)^18^.

In the relevant period, digital Community Inclusion Currencies (CICs) were technologically accessible to most of the Kenyan population. The USSD interface requires a phone number (i.e. a SIM card) and access to a mobile phone. Per nationally representative surveys, upwards of 86% of Kenyans (age 15+) owned a SIM card and over 90% owned or had access to a mobile phone^16,55^. The top reason given for not owning a mobile phone was not having enough money to buy one. GE targets marginalized, food insecure areas of Kenya where digital inclusion is somewhat lower (see Targeted Introductions).

### Record keeping

The Sarafu system is administered by GE via a custom-built CIC management platform (see Code Availability). During the period from January 25, 2020 to June 15, 2021 all system activity was centrally recorded in a PostgreSQL database. SQL stands for “Structured Query Language” and is a standard format for organizing linked tables into a database containing, in this case, account and transaction information. There were four processes that generated the stored records: account creation, USSD transactions, currency management, and administrative tasks.

When possible, transactions from the PostgreSQL database were duplicated onto the xDAI blockchain. The xDAI blockchain is public by design, and transactions recorded on it cannot be reversed. This served as a secondary repository for this data in this period. The majority of transactions also appear there; any gaps were due to outages and fees. Blockchain integration was simple, in this case, as only Sarafu digital tokens were used within the system we describe over the observation period. The token_address for Sarafu on the xDAI blockchain is included for the benefit of researchers studying cryptocurrencies. Blockchains offer additional functionality for CICs that may be incorporated in the future^56^.

#### Account creation

During the observation period, individuals could create an account and join the Sarafu system by dialing a USSD short code and setting a personal authentication code (PIN). This would establish a new account tied to their phone number, secured behind their PIN. Staff at GE could be reached by phone to provide assistance. Newly-created accounts were given an anonymous wallet address on the xDAI blockchain as a unique identifier.

New users could opt into supplying their name, gender, and “home location” via the USSD interface. Users could also choose to describe the goods or services they provide to the community and intend to exchange for payment in Sarafu, the “product category”. Personally identifying information, including name and phone number, is kept confidential (see Anonymization). The user-generated entries for home location and product category can be overly precise and were generalized into broader categories by GE using a coded mapping (see Code Availability). These are area_name and business_type in the user data. Note that account information could be updated or removed at any time. This paper describes the snapshot of account data retrieved on June 15, 2021.

Newly created accounts are assigned the held_role of beneficiary, by default. Indeed, most accounts are held by an individual. There was a process in place to verify individuals’ account information (see the Currency management section, below). Nevertheless, multiple accounts, account sharing, spam accounts, and other “off-label” uses may be present to some extent. The data also contains a number of accounts with a group_account designation. These belong to local groups – i.e. chamas – whose community operations have been verified by GE. Chamas are usually composed of 15–30 people, often defined by a neighborhood, a shared occupation, or friendship and family ties^15,17,57^. Field staff would help ensure that chama accounts were properly administered, locally, often by the group’s treasurer. The recorded user information sometimes reflects this designated administrator. Note that an account’s role is not necessarily static and that chamas themselves are not always permanent; account designations may have been changed at some point prior to June 15, 2021.

#### USSD transactions

**Fig. 1:**
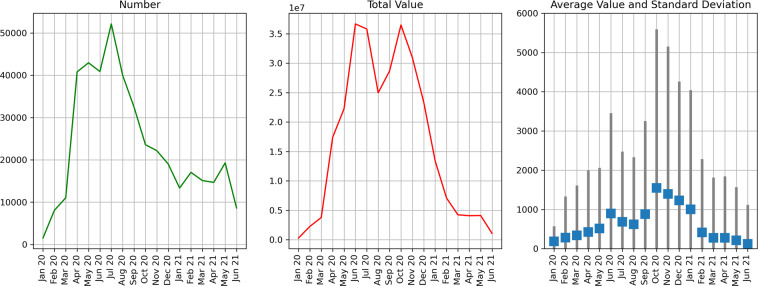
standard transactions. Monthly time series showing the number, total value, and average value of standard transactions.

Transactions made via the USSD interface over the observation period were automatically recorded in the accounting database. In these records, the accounts that sent and received the transaction (source and target in the transaction dataset) are referred to by their xDAI blockchain address. Also noted is the timestamp of the transaction and its amount in Sarafu (timeset and weight in the transaction dataset). The vast majority of transactions made via the USSD interface during this period were ordinary transfers among users, noted as transfer_subtype standard. Figure 1 shows the monthly transaction volume of such transactions. For elaboration on time trends we refer to Usage Notes and Section 4.1 of Ussher *et al*. (2021)^18^.

Between January and June 2020, a limited cash exchange functionality was also available (see Technical Validation). Chamas that met particular criteria could exchange a portion of their Sarafu balance for Kenyan Shillings (see Usage Notes). During such an operation, a group_account would send Sarafu to a special account holding the role token_agent via the USSD interface; such transactions were recorded as type agent_out.

#### Currency management

Currency creation and dissolution in the Sarafu system took place via disbursement and reclamation type transactions, respectively. Currency management includes:

- disbursing Sarafu tokens to new users,
- sending a bonus to those who verified account information with GE staff,
- rewarding the first transaction in Sarafu with a new user,
- rewarding high levels of transaction activity in Sarafu,
- penalising large balances held in Sarafu, and
- promoting wider use of Sarafu.

These functions were partially automated during the data collection period; batch scripts and manual operations were also used. Note that 2020–2021 was a period of transition and experimentation for CICs and so currency management protocols were modified at times and not always consistently applied (see Supplementary File 1).

##### Initial disbursement

Newly opened accounts received an initial allotment of Sarafu via a disbursement transaction. Early on, the amount that new users received was 400 Sarafu. On May 1, 2020 initial disbursements were fully automated and reduced to 50 Sarafu. Accounts existing prior to January 25, 2020 received initial funds separately (see Administrative tasks).

##### Verification bonus

Grassroots Economics provides training for new users and encourages new users to contact the phone support team. Staff also reach out via phone to verify account information and provide training to new users. Speaking with GE prompts a verification bonus, sent as a disbursement transaction. Early on, these were valued at 250 or 100 Sarafu and processed in groups. Beginning May 1, 2020, these bonuses were processed manually, almost daily, with disbursements valued at 350 Sarafu. On October 12, 2020 the verification bonus was reduced to 50 Sarafu. Most new users in this period were eventually verified, so most new accounts received a verification bonus in addition to their initial disbursement (i.e., initial disbursements plus verification bonus such as 400 + 250, 400 + 100, 50 + 350, or 50 + 50).

Between January and June 2020, verified chamas also received bonuses. The group_account itself would receive an extra disbursement transaction of 10,000 Sarafu or the members of a verified chama would receive a total of 10,000 Sarafu. Bonuses via chamas were phased out and had ended by July 2020, when support for chamas changed form (see Usage Notes).

##### Weekly rewards

Sustained transaction activity is considered key to the sustainability of a community currency, and GE incentivized particular kinds of activity with weekly rewards in Sarafu. The definition of active varied, but generally implied a minimum of one transaction in a week. From March 2020, users were regularly rewarded in proportion to particular metrics calculated over the week or so before (e.g., number of trading partners, number of transactions, clustering coefficient). Active accounts received a disbursement with the reward amount. Beginning April 14, 2020, weekly rewards were administered in batches with amounts up to a few hundred Sarafu; most active users received rewards with a value below 100 Sarafu. Beginning December 2020, each user who made at least one transaction in the preceding week was awarded the same amount. Uniform weekly rewards ranged from 20 to 50 Sarafu until February 16, 2021 when they were standardized to 30 Sarafu.

##### Referral rewards

Integrating new users into the transaction network is considered key to promoting community adoption of Sarafu. Existing users were incentivised to trade with new users: the first person to accept payment in Sarafu from a new user was rewarded. That is, the first account that receives a standard transaction from a newly-created account was also to receive a disbursement transaction. Referral rewards were 100 Sarafu for each person referred and processed manually alongside initial disbursements (from January through April) or verification bonuses (from May through September). Beginning September 21, 2020, referral rewards were often combined with the weekly reward. On October 12, 2020, the amount was reduced to 50 Sarafu per referral. Note that Grassroots Economics identified a few instances of the deliberate misuse of the referral rewards incentive, which resulted in some reclamations, warnings, and account suspensions.

##### Demurrage charges

Eventually, Grassroots Economics disincentivized the hoarding of Sarafu tokens by charging a demurrage fee on the balance held by users. Demurrage charges were levied in batches of reclamation transactions, with the fee proportional to the account balance. Demurrage was tested in November 2020 at a weekly interval (at a 0.5% rate) and then charged at a regular monthly interval (at a 2% rate) beginning February 1, 2021. Note that batches of demurrage reclamations can be large; these were prepared together and then processed by the system, one at a time, sometimes over several days.

##### Promotional bonus

Promotions in the form of broad-based bonuses took place four times during this period. In each case, the same amount was disbursed to most (or all) Sarafu accounts. Promotional bonuses were large batch operations, sometimes combined with rewards or charges. Table 1 describes these operations.

**Table 1:** Broad-based promotional operations.

Date	Amount	Combined with
April 30, 2020	100	None
November 2, 2020	100	Weekly, Referral, Demurrage
December 28, 2020	50	None
March 22, 2021	10	Weekly, Referral

#### Administrative tasks

Grassroots Economics maintains a number of staff-run accounts for completing ad-hoc tasks required in the administration of Sarafu as a digital community currency. These include:

- Sarafu was consolidated into a single CIC on January 25, 2020 (as mentioned in the Background & Summary section). This change was effected as follows: users of precursor currencies received a wallet address on the xDAI blockchain and were sent a disbursement transaction of Sarafu equivalent in size to their balance held in prior wallets, at a 1:1 conversion rate. Note that prior wallets for the precursor currencies were on the now-defunct POA blockchain.
- Before regular demurrage charges were introduced, ad-hoc penalties were assessed on inactive accounts. Inactive users were charged a small fee of (up to) 20 Sarafu on two occasions in April 2020. On October 19, 2020, substantial penalties were assessed on predominantly long-inactive accounts; many were zeroed out.
- Staff-run accounts were used to correct mistakes introduced by users, staff, and technical glitches. Since erroneous transactions cannot be reversed, mistakes were often resolved with a disbursement/reclamation pair.
- Verified chamas were eligible to receive donations via GE. Staff-run accounts with held_role admin or business_type *system*, and the account with held_role vendor (an e-commerce wholesaler) have been used to distribute donations in Sarafu and in kind. For a detailed description of support to chamas, see Usage Notes.
- During the observation period, Sarafu was also a part of particular development and disaster-response interventions. For a detailed description of known interventions and the traces they leave in the data, see Usage Notes.

### Anonymization

The identity of Sarafu users is to be kept confidential. To mitigate disclosure risk, a series of modifications were made to the user dataset. Table 2 summarizes the steps taken as per UK Data Service guidelines on anonymization and data sharing. Recall that users are given an anonymous unique identifier during the routine administration of the Sarafu system. Also, user-generated fields are standardized and aggregated. In extracting the data from the PostgreSQL database all personally identifying information and idiosyncratic user-generated fields were excluded. In post-processing, remaining counties with few Sarafu users were merged and re-coded.

**Table 2:** Disclosure risk mitigation steps.

Variable	Disclosure risk	Mitigation
Phone number	Personal identifying information	Excluded from dataset
Home location	Overly precise responses	Aggregated into standard codes as area_name
Product category	Overly precise responses	Aggregated into standard codes as business_type
Area name	Low number of Sarafu users in county	Recoded into a broader category

The transaction dataset references only the anonymous identifiers and these records do not contain personally identifying information; the xDAI blockchain is public by design. Still, in general, digital trace data is somewhat sensitive in that high-resolution records could conceivably be linked to outside information on recorded activity^58^. This residual risk of disclosure is mitigated by appropriate limitations placed on access and reuse of the dataset (see Usage Notes), and by the secure environment in which non-anonymous activity takes place. Disclosure of transaction histories by account holders is against the Sarafu Network Terms of Service (https://www.grassrootseconomics.org/pages/terms-and-conditions.html).

## Data Records

The Sarafu CIC 2020–2021 data^13^ consists of two tables, the “transaction dataset” and the “user dataset”. Here we describe the content and format of each table.

### Transaction dataset

The transaction dataset includes a record of every transaction executed in Sarafu from January 25, 2020 to June 15, 2021. The transaction dataset is stored in the sarafu_txns_20200125–20210615.csv file. Each record corresponds to a transaction made within the Sarafu system, including the following columns:

- id — *Assigned by the system*. PostgreSQL transaction index.
- timeset — *Assigned by the system*. Date and time of transaction. Format: YYYY-MM-DD HH:MM:ss.ms
- transfer_subtype — *Assigned by the system*. The standard transactions are regular transfers made via the USSD system. A disbursement transaction creates Sarafu and adds them to an account. Analogously, reclamation transactions remove Sarafu from an account. agent_out transactions facilitate cash exchange operations, where donors bought Sarafu from chamas using Kenyan Shillings.
- source — *Assigned by the system*. Wallet address of the user that sends the transaction.
- target — *Assigned by the system*. Wallet address of the user that receives the transaction.
- weight — *Assigned by the system*. Amount transferred from source to target, in units of Sarafu.
- token_name — *Assigned by the system*. In this period, this was fixed as ‘Sarafu’.
- token_address — *Assigned by the system*. Blockchain code of Sarafu.

### User dataset

The user dataset reflects the information about account-holders as of June 15, 2021. Values in the dataset are final, seeing as people may have updated their information or moved since the account was created. The user dataset is stored in the sarafu_users_20210615.csv file. Each record corresponds to a registered account with the Sarafu system, including the following columns:

- id — *Assigned by the system*. PostgreSQL user index.
- start — *Assigned by the system*. Date of account registration. Format: YYYY-MM-DD HH:MM:ss
- final_bal — *Assigned by the system*. Final balance in units of Sarafu at the time the dataset was extracted.
- gender — *Declared by the user*. The user can select among *Male, Female*, and *Other* via the USSD interface. Missing values are *Unknown*.
- area_name — *Assigned by GE staff*. Standardized name of a geographic area derived from the user-provided home location: *Kinango Kwale, Mukuru Nairobi, Misc Nairobi, Kisauni Mombasa, Misc Mombasa, Kilifi, Nyanza, Turkana, Misc Rural Counties, other*. Missing values are included in *other*.
- area_type — *Assigned by GE staff*. Designation of settlement type based on user-provided home location: *urban, rural, periurban, other*. Missing values are included in *other*.
- held_roles — *Assigned by the system*. Designation of the account type as recognized by the system. beneficiary accounts are regular users; this is the default. group_account designation indicates a recognized local savings and investment group (chama). admin accounts are used by staff for currency management and administrative operations. The token_agent and vendor accounts were used to facilitate in-cash and in-kind donations, respectively. ‘None’ accounts were created in error and are not tied to a user.
- business_type — *Assigned by GE staff*. Standardized category of economic activity derived from user-provided product category. A description of the values:
  - *labour* — Non-farm workers.
  - *food* — Sellers of local food.
  - *farming* — Farmers or workers on farms, e.g., labourers specialised in plowing fields.
  - *shop* — Kiosks, boutiques, phone shops, cafes, pubs, clothing shops, home furniture shops, flower shops, etc.
  - *fuel/energy* — Sellers of firewood, kerosene, petrol, biogas, charcoal, paraffin, and diesel.
  - *transport* — Drivers, bicycle, motorbike, and car services. Boda boda riders carry clients on a motorbike.
  - *water* — Water re-sellers and people managing water storage equipment.
  - *education* — Teachers in schools and day cares. Coaches, tutors, etc. Red Cross volunteers and demo accounts.
  - *health* — Traditional and official doctors, nurses, pharmacies, laboratories, veterinarians.
  - *environment* — Waste collection, gardening, seeding, tree planting, cleaning, recycling.
  - *savings* — Savings and investment groups, chamas, sometimes members thereof.
  - *government* — Community authorities (e.g. elders), government employees, local and military officials, soldiers.
  - *faith* — Pastor, imam, counsellor, etc.
  - *system* — Account managed by Grassroots Economics staff.
  - *other* — Unknown

- xDAI_blockchain_address — *Assigned by the system*. Wallet address on xDAI blockchain; unique ID.
- old_POA_blockchain_address — *Assigned by the system*. Wallet address on the now-defunct POA blockchain. This variable is blank unless the account existed prior to January 25, 2020.
- ovol_in/ovol_out — *Computed by GE*. Total volume coming into or out of the account via operations involving GE, in units of Sarafu.
- otxns_in/otxns_out — *Computed by GE*. Number of transactions into or out of the account via operations involving GE.
- ounique_in/ounique_out — *Computed by GE*. Unique contacts into or out of the account via operations involving GE.
- svol_in/svol_out — *Computed by GE*. Total volume coming into or out of the account via regular activity, in units of Sarafu.
- stxns_in/stxns_out — *Computed by GE*. Number of transactions into or out of the account via regular activity.
- sunique_in/sunique_out — *Computed by GE*. Unique contacts into or out of the account via regular activity.

## Technical Validation

This section presents analyses supporting the technical quality of the dataset. We demonstrate the precision of the the accounting over the observation period and confirm that key currency management operations are correctly described. User characteristics reflect expectations gathered from relevant field studies.

### Accounting consistency

Here we validate the accounting consistency of the entire transaction dataset against the account balances recorded in the user dataset on June 15, 2021. Ideally, we should be able to obtain each account’s final_balance by netting their inflows and outflows over the time period. Table 3 provides a summary of the records used to compute the expected total balance.

**Table 3:** Summary of transaction dataset.

Transfer subtype	Total number	Total value
standard	422,721	296,991,020
disbursement	308,425	36,299,907
reclamation	198,745	16,884,943
agent_out	270	6,172,075
total	930,161	356,347,944

The accounting discrepancy between the user dataset and the transaction history is 3,271 Sarafu, or 0.017% of the system balance on June 15, 2021. In the observed period, the total inflow to beneficiary accounts was 265,639,898 Sarafu and the total outflow was 250,809,052 Sarafu. The group_accounts recorded a total inflow of 63,019,042 Sarafu and a total outflow of 58,738,067 Sarafu. The expected total balance was 19,419,736 Sarafu, calculated as the inflow minus the outflow for all the accounts (excluding held_role admin). The sum of the final balances reported in the user dataset was 19,416,465. The difference between the two arises out of a mismatched balance for 80 users, or 0.16% of accounts. This is remarkably precise, as only 0.00091% of the total transaction volume appears to have been misrecorded.

### Initial disbursements

Newly created accounts receive an initial disbursement transaction. Hence, we expect to observe similar trends in the timeseries of “initial disbursements” and in the creation of new accounts. As such, we compare the registration dates of new accounts (user dataset: start) with the timing of disbursement transactions matching the criteria for an initial disbursement. These are described in the Currency management section and a provisional labeling is provided in Supplementary File 1. We exclude the accounts inherited from precursor currencies at the start of the data (user dataset: old_POA_blockchain_address).

**Fig. 2:**
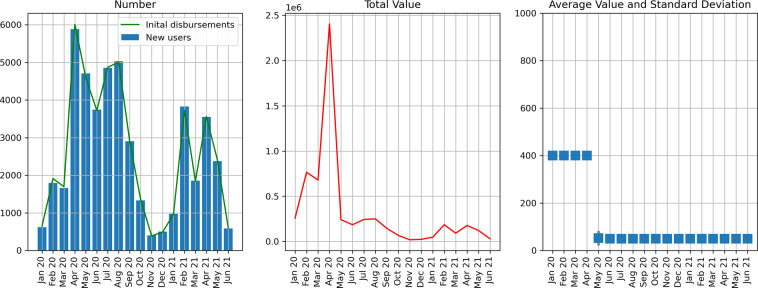
Initial disbursements. Monthly time series showing the number, total value, and average value of initial disbursements. The leftmost panel also shows the number of newly registered Sarafu users, for comparison.

Figure 2 shows the number of account creations and initial disbursements over time; there is a tight correspondence. Note also the change in the value of initial disbursements in May 2020 and its effect on the total value dispersed. Over the entire observation period, initial disbursements made up 15.14% of all disbursement transactions and 16.36% of the total value of Sarafu created.

### Demurrage charge as 2% holding fee

Standardized demurrage has been charged monthly since February 2021. This is done via a batch operation where the balance of each account is queried, the 2% holding fee is calculated, and a reclamation transaction is initiated against each account. Here we validate the February 1, 2021 demurrage operation by computing the balance of each account on January 31, 2021 as per prior transaction records. The expected fee to be charged of each account is 2% of the balance (in this case, rounded). The total value of expected fees is 352,806 Sarafu, and the total value of reclamations over February 1–3, 2021 was equal to 352,834. This is a difference of only 28 Sarafu, or 0.008% of the demurrage charged.

### Cash exchange operations

“Cash exchange” operations were used until July 2020 to distribute cash donations to chamas that met particular criteria (see Usage Notes). agent_out transactions facilitated the exchange of Sarafu for Kenyan Shillings, and the Sarafu funds were dissolved. Recall that agent_out transactions were made from a group_account to the token_agent. To confirm that agent_out transactions reflect “cash exchange” operations that removed Sarafu from the system, we compare the amount of Sarafu sent to the token_agent account as agent_out transactions against that collected from the token_agent account via reclamation transactions. These values are within 0.02% of one another (6,172,075 Sarafu and 6,173,351 Sarafu, respectively).

### User characteristics

**Fig. 3:**
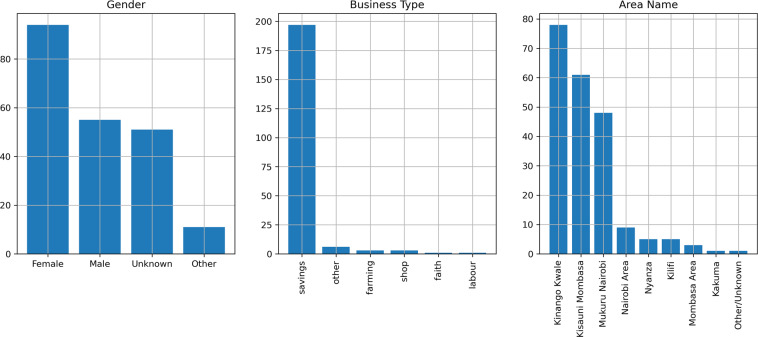
Characteristics of group_accounts. Bar plots detailing the number of group_accounts with each observed value for gender, business_type, and area_name.

**Fig. 4:**
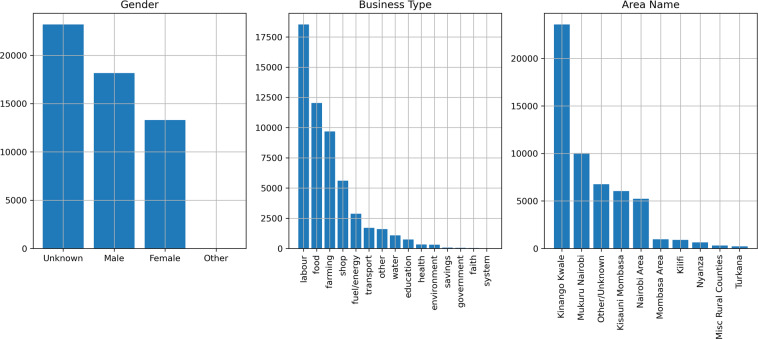
Characteristics of beneficiary accounts. Bar plots detailing the number of beneficiary accounts with each observed value for gender, business_type, and area_name.

Participation in the Sarafu system is voluntary, so this data does not reflect a sample of any particular population. For this reason, we validate the reported user characteristics against prior fieldwork studying the Sarafu system itself, specifically. We expect to observe particular patterns as highlighted in theses, case studies, and other qualitative reports^11,15,17,57^. Figures 3 and 4 show the gender, business_type, and area_name for the group_account and beneficiary accounts, respectively. The reported user characteristics broadly reflect expectations:

- In the relevant communities, many chamas have predominantly women participants^17^. We find that 94 group_accounts (45%) declare the group’s designated administrator as *female* and only 55 (26%) as *male*.
- Recognized chamas are primarily groups used for pooling, investing, and lending savings. Indeed, the vast majority of the 211 group_accounts (93%) have business_type *savings*.
- The most common business_type assigned to the activities reported by beneficiary accounts are forms of self-employment: offering labour (34%), selling wares (10%), and participating in farming (18%) or food distribution (22%). This is consistent with patterns of employment in marginalized, food insecure areas^11^.
- The reported location often corresponds to an area_name where GE has a substantial presence (Kinango Kwale) or targeted introductions were taking place (Mukuru Nairobi & Kisauni Mombasa). Further context is provided in Usage Notes. This expected spatial distribution is especially pronounced for group_accounts, as this designation relies on the availability of field staff (see Account creation in Methods).

## Usage Notes

The Sarafu CIC 2020–2021 data is available via the UK Data Service ReShare platform (https://reshare.ukdataservice.ac.uk/855142/)^13^. The data is “safeguarded”, meaning that access is limited to those who have registered with the UK Data Service (UKDS) and allowable reuse is constrained by their End User License (EUL). To register is to agree to abide by the conditions of the EUL or risk revocation of access to UKDS and potential legal action. In particular, the datasets must be kept secure and must not be used to attempt to identify individuals in the data. Use of the data for research that aims to de-anonymize or re-identify Sarafu users is expressly prohibited. Grassroots Economics (GE) has made this data available for research under these privacy-preserving stipulations as a part of its broader mission, in keeping with applicable laws and regulations. Describing and analyzing the published data does not require the approval of the Ethical Review Committee (ERC) according to the Leiden University Faculty of Science ERC, provided the research team maintains compliance with the privacy-preserving stipulations governing re-use.

Future researchers may find it desirable to consider also data from other sources, such as official statistics, public health surveillance, or representative surveys (e.g., FinAccess^16^ or Financial Inclusion Insights^55^). Indeed, doing so would enable research into digital and financial inclusion^28–32^, informal credit and insurance arrangements^15,26,33,34^, economic growth and disruption^3,4,18^, and other relevant topics in development. The values of area_name provided in the user dataset generally correspond to past or present administrative areas in Kenya, making linkage possible. However, Sarafu users do not reflect a statistical sample of the population of these areas—activity is concentrated in specific places and outreach efforts have been aimed at marginalized, food insecure communities. Reproducing the geographic distribution of Sarafu users may be possible, to an extent, using details included within the data extraction and processing code (see Code Availability). Specifically, the file sarafu_user_db_test.py contains the mappings by which user-defined fields were standardized and aggregated. This includes the set of (often colloquial) local place names reported by at least one Sarafu user, which could perhaps be geocoded. Again, aiming to de-anonymize or re-identify Sarafu users is expressly prohibited. Looking forward, research designs that combine digital traces with complementary data, such as surveys or financial diaries, already at the data collection stage are methodologically promising^58–62^.

The remainder of this section describes the context in which Sarafu operates as a community currency—Community Inclusion Currencies (CICs) are considered a promising development intervention and the observation period includes the first year of the COVID-19 pandemic. Early community currencies implemented in Kenya by the founders of GE encountered some success in stimulating local economic activity^10,63^ and Sarafu has come to be seen as a CIC pilot project^64^. Indeed, the underlying technology is openly available to facilitate the implementation of CICs in other areas (see Code Availability). Recruitment campaigns, called targeted introductions, have shaped patterns of adoption and use. Beginning in March 2020, Sarafu was also made part of a humanitarian response effort to the emerging COVID-19 pandemic^65–67^. Chamas played a prominent role as the recipients of direct donations. We also describe specific development, disaster-response, and experimental interventions undertaken by GE and others during the observation period, providing citations to additional resources on these initiatives.

### Targeted introductions

Patterns in the adoption and use of Sarafu during this period are strongly shaped by past and present initiatives aimed at introducing the complementary currency to targeted communities. GE has a history of conducting such campaigns in collaboration with partner organizations^11,18^. This section provides reference material for targeted introductions, past and present. We also detail a notable example of a community for which Sarafu serves as a complementary currency.

Grassroots Economics (since 2015) and its founders (since 2011) have introduced community currencies into informal settlements of Nairobi^11,12,68^ and Mombasa^10,12,69–72^ as well as communities in periurban Kilifi and rural Kwale^11,57^. Recall that Sarafu is the union of 12 distinct CICs, each of which had its own digital token until January 2020 (see Background & Summary). Ussher *et al*.^18^ provide a richer history of GE up to and including the data collection period. Marion^11^ lists the longest-running community currencies that joined the Sarafu system. User activity in these areas continued to be supported by GE through the observation period.

During the observation period, there were two targeted introductions of note. Each of these was a partnership between GE and the Kenyan Red Cross. In April 2020, the Red Cross began educational and outreach programs in a village in the Mukuru kwa Njenga slum of East Nairobi. While this pilot project was long-planned, the timing meant that it soon became an improvised response effort to the emerging COVID-19 pandemic. Promotion, education, and training were held in the field by Community Based Disaster Response Teams (CBDRTs) managed by the Red Cross^64,65,73^. Note that the volunteer members of Red Cross CBDRTs received “initial disbursements” and often also “referral rewards”, serving as a form of compensation. In early 2021, a second pilot project by the Kenyan Red Cross introduced Sarafu in Kisauni, Mombasa^67,74^. Ussher *et al*.^18^ offer relevant analyses of time trends, with breakdowns of transaction activity by area_name.

While targeted introductions leave traces in the spatial distribution of Sarafu users, much of Sarafu’s expansion over the observed period was due to word-of-mouth^66^.

#### Kinango Kwale

Grassroots Economics has a substantial presence in areas of Kinango Kwale. The local economy faces three major risks: seasonal food scarcity, climate-driven variability, and volatility in nearby urban markets. According to the Kenyan Ministry of Agriculture, about 70% of households in the county of Kwale are food poor and 14% do not have regular access to food^75^. The typical pattern of rainfall in Kenya has a “long rains” season from March to May, a “short rains” season from October to December, and two drier periods. Food insecurity peaks between April and June. The sub-county of Kinango is considered semi-arid; climate-driven variability and extreme weather events are increasing^75^. Poverty and low agricultural productivity has forced many to start small businesses besides farming or to look for work in nearby urban areas^11^.

Community development initiatives and the operation of the Sarafu system in this area are documented in fieldwork-based theses^11,57^ and other recent studies^18,76^. The approach GE takes is best illustrated by providing an example: in one cluster of villages, efforts began in support of an existing agricultural and community-based cooperative set up by the Kenyan Red Cross, the Kenyan Government, the Green World Campaign, and the World Food Program. A physical community currency was established in 2017 with the aim of bolstering local markets. GE also made targeted investments in the area aimed at ensuring the circulation of the community currency (e.g., maize milling, refrigeration, and water storage equipment). Support was provided for community-based income and investment activities (e.g., chamas). The community currency was digitized in early 2019 via registration drives run by field staff, and became part of the unified Sarafu CIC on January 25, 2020. During the observation period, the area received direct support via donations to chamas and was a target for investments in social enterprise development, as described below.

### Donations to chamas

Grassroots Economics rewarded chamas who bolstered participation in the Sarafu system. Donations from the DOEN Foundation and the Danish Red Cross were routed directly to local communities via top chamas, in cash and in kind. With the arrival of COVID-19 and attendant economic disruptions in Kenya, donations also took on the role of pandemic aid. The manner of support for chamas, and thus the mechanisms used to distribute donations, evolved over the course of the year 2020:

- Between January and July 2020, chamas with sufficient trade volume could exchange up to half of their balance for cash. Donor funds were used to purchase Sarafu from chamas in Kenyan Shillings (up to 30,000 Sarafu, monthly). This was done primarily via “cash exchange” operations, but sometimes also as ad-hoc reclamation transactions and as standard transactions to an account with business_type *system* (id 184).
- Between August and December 2020, a food distribution program was in place where top chamas could use Sarafu to purchase food from local vendors of their choice. The amount chamas could purchase was based on metrics of Sarafu CIC usage. Groups would pay in Sarafu to the id 184 *system* account or to an account with held_role vendor. Later, GE would compensate the vendors in Kenyan Shillings.
- Direct donations via Sarafu were entirely phased out towards the end of 2020. Instead, top chamas would simply receive farm inputs or digital payments in Kenyan Shillings; support for chamas no longer leaves direct traces in the Sarafu data.

### Specific interventions

The Sarafu system has also been used for specific interventions, at particular times and in particular places. Here we describe three interventions undertaken by GE in collaboration with other organizations in Kenya. We identify the traces the relevant operations left in the data itself, and offer citations to more detailed accounts of the interventions.

#### Social enterprise development

Grassroots Economics has continued to make targeted investments in local economies that encourage the circulation of Sarafu within the community. During the observation period, selected chamas received support for developing cooperative income-generating activities linked to Sarafu acceptance. Chamas were selected by GE and partners based on CIC usage, membership size, and locality. Examples of initiatives include urban sack gardens and a program in partnership with Mustardseed Trust to establish community farms that practice regenerative agriculture^77^. Large disbursement and/or reclamation transactions in the data can indicate targeted investments; later in the observation period these were no longer administered via Sarafu and are thus not visible.

#### Disaster-response intervention

Disruptions related to the COVID-19 pandemic arrived in Kenya in March 2020. The targeted introduction of Sarafu into the Mukuru kwa Njenga slum in Nairobi came to be seen by the Red Cross as a disaster-response intervention^65,66^. An impact evaluation survey was run in May 2020^67^. While outreach and education were the Red Cross’ main focus, specific pandemic-related efforts also took place. A local wholesaler donated health kits, and hygiene items were distributed to chamas, shops, and local clinics in part via payment in Sarafu. The recipient of such payments was one of the accounts with business_type *system*, specifically id 184. During March and April of 2020 a cash-in promotion was active; uptake was very limited with small amounts disbursed to a handful of users. The country saw several peaks in cases of COVID-19 in 2020–21.

#### Randomized controlled trial

Mqmelo (2021) presents the results of one of the first randomized controlled trials on community currencies^78^. From November 20, 2020 to December 4, 2020, a treatment group of beneficiary accounts received 400 Sarafu over three consecutive weeks. The dataset includes 399 apparently treated users in Nairobi and 134 in Kinango Kwale. This experiment was approved by the Minerva University Human Subjects Research (HSR) committee.

## Supplementary information

Supplementary File 1

Supplementary File 2

## Data Availability

The software underlying Community Inclusion Currencies and the Sarafu implementation are available in public repositories at https://gitlab.com/grassrootseconomics. The data extraction and processing code described in the Anonymization section is available in a separate public repository at https://github.com/grassrootseconomics/dashboard (especially: sarafu_user_db_test.py). The script invoked by staff at GE to run the data extraction and processing code is included in the documentation folder that accompanies the dataset via the UK Data Service ReShare repository^13^. Supplementary File 1 is a code notebook that provides a provisional labeling for disbursement/reclamation transactions and a procedure for noting known artefacts in the data. Supplementary File 2 reproduces the technical validation.
